# Identification of cell senescence-related genes in spontaneous preterm birth based on bioinformatics analysis and machine learning

**DOI:** 10.1371/journal.pone.0340809

**Published:** 2026-01-16

**Authors:** Guo Juan, Li Ting, Wang Yahui, Zuo Luguang

**Affiliations:** Clinical Laboratory, The First Affiliated Hospital of Hebei North University, Zhangjiakou City, Hebei Province, China; University of Arkansas for Medical Sciences, UNITED STATES OF AMERICA

## Abstract

Spontaneous premature birth (SPTB) is a common pregnancy complication; however, few studies have explored cell senescence-related markers in SPTB. Bioinformatics and machine learning approaches were used to predict potential biomarkers associated with SPTB. Normal and SPTB gene expression profiles were obtained from the Gene Expression Omnibus (GEO) database, and cell senescence-associated genes from the Human Aging Genomic Resources (HAGR) database. Functional enrichment analysis and protein-protein interaction (PPI) network analysis of differentially expressed senescence-related genes in SPTB were conducted using Gene Ontology (GO), Kyoto Encyclopedia of Genes and Genomes (KEGG), and STRING databases. The infiltration of 22 types of immune cells in SPTB was calculated using the CIBERSORT deconvolution algorithm. Machine learning methods were employed to identify hub differentially expressed genes (DEGs). Datasets GSE174415 and GSE118442 were extracted for validation to determine the final hub genes. Additionally, receiver operating characteristic (ROC) curves were constructed to assess the diagnostic potential of the hub genes, and significant pathways associated with the final hub gene were explored by Gene Set Enrichment Analysis (GSEA). Finally, real-time quantitative polymerase chain reaction (RT-qPCR) was performed to validate the hub gene in clinical specimens. A total of 923 DEGs were identified, including 525 upregulated and 398 downregulated in the SPTB group. These 923 genes were intersected with 866 cell senescence-related genes, yielding 48 intersection genes. Functional enrichment analysis indicated that these intersection genes were primarily associated with cytokine–cytokine receptor interactions and the PI3K-Akt signaling pathway. The expression of activated dendritic cells and follicular helper T cells was significantly lower in the SPTB group compared to the full-term pregnancy group. A total of six hub genes, LGALS3, ESR1, PLA2G2A, TWIST1, CBS, and PLA2R1, were identified by machine learning. According to dataset validation, TWIST1 was identified as the final hub gene. TWIST1 was downregulated in placental tissues of the SPTB group and demonstrated high diagnostic value for SPTB. Thus, TWIST1 may be a novel molecular target for predicting and diagnosing SPTB, providing diagnostic value and novel insights into this condition.

## Introduction

Preterm birth is defined as delivery between 28 and 37 weeks of gestation [1]. Based on its causes, preterm birth can be divided into SPTB and therapeutic preterm birth. SPTB includes spontaneous preterm labor and preterm premature rupture of membranes (PPROM) and accounts for approximately 70%–80% of preterm births [2]. Preterm birth is an important cause of death and disability among infants and children under five years, affecting nearly 15 million infants annually and contributing to over a million infant deaths [3]. China’s preterm birth rate is at a global medium level, approximately 6.70% [4]. However, due to lifestyle changes, an increased number of older pregnant women following birth policy adjustments, altered pregnancy intervals, and a higher incidence of pregnancies complicated by medical and surgical conditions, the incidence of preterm birth is rising [5]. Consequently, China’s preterm birth rate now ranks second globally [6]. The large number of premature infants, their poor survival rates, and high mortality impose heavy burdens on families and society [7]. SPTB is a complex, multi-etiological syndrome. Numerous factors contribute to SPTB, but there remains no consensus on the underlying mechanisms [8]. Identifying the pathogenesis and development of SPTB is therefore essential for early prevention and reducing premature infant mortality and long-term disease risks.

The proper formation and function of the placenta are essential for establishing and maintaining pregnancy. Trophoblast cells represent a crucial component of the placenta, necessary for embryo implantation and placental development [9]. Placental senescence is defined as the gradual decline in placental function with advancing pregnancy, usually occurring in the third trimester, and is a normal physiological phenomenon [10]. Cellular senescence includes replicative senescence and premature senescence and serves as an important cellular defense mechanism. Various external environmental stimuli or internal stress factors can trigger this process, eventually leading to irreversible cell cycle arrest. Cellular senescence is a complex biological phenomenon involved extensively in regulating organismal homeostasis. It plays a critical role in ontogeny, tissue homeostasis maintenance, and the pathogenesis of age-related diseases [11]. Physiological cellular senescence occurs during decidualization and placental development. However, pathological decidualization of stromal cells and placental trophoblast cells induced by various stimuli can result in adverse pregnancy outcomes [12].

In this study, bioinformatics and machine learning methods were applied to explore molecular markers related to cellular senescence in SPTB and to investigate the role of hub genes. Through comprehensive analysis of gene expression profiles and regulatory networks associated with cellular senescence in SPTB, the role of cellular senescence in the occurrence and progression of SPTB was clarified. This approach provides novel molecular targets for the prediction, diagnosis, and treatment of SPTB.

## Materials and methods

### Collation of cell senescence-related genes

Human senescence-related genes were obtained from the Human Aging Genomic Resources (HAGR, https://genomics.senescence.info/) database [13].

### Screening and analysis of differential expressed genes

High-throughput sequencing datasets associated with SPTB were retrieved from the GEO database (https://www.ncbi.nlm.nih.gov/geo/) using “spontaneous preterm birth” as keywords. The GSE174415 dataset, containing 16 SPTB samples and 16 full-term pregnancy placenta samples, was selected as the training set. The R package DESeq2 was employed to compare gene expression levels between the two groups and screen for DEGs. Screening criteria were |log2FC| > 0.585 and P < 0.05. The identified DEGs were visualized using ggplot2, pheatmap, and ggVolcano packages. The VennDiagram package was used to determine intersection genes between the DEGs and cell senescence-related genes.

### GO and KEGG enrichment analysis

GO functional enrichment analysis and KEGG pathway analysis were performed using the ClusterProfiler package in R [14].

### PPI network analysis

The PPI network of intersection genes was constructed using the STRING database (https://string-db.org) with a confidence threshold of 0.40. Results were exported to Cytoscape software for further analysis and visualization [15].

### Immune infiltration analysis

The CIBERSORT algorithm utilizes linear support vector regression (SVR) to deconvolute the expression matrix of 22 human immune cell subtypes [16].To investigate immune cell infiltration in SPTB, the proportions of 22 immune cells in the GSE174415 dataset were calculated using the CIBERSORT deconvolution algorithm through the R package. Histograms and boxplots were generated using the ggplot2 package in R. Pearson correlation analysis was performed to investigate the correlation between TWIST expression level and the proportions of immune cell infiltration.

### Machine learning for screening hub genes

Least absolute shrinkage and selection operator (LASSO), support vector machine recursive feature elimination (SVM-RFE), and eXtreme Gradient Boosting (XGBoost) algorithms were applied to identify cell senescence-related genes in SPTB. For LASSO regression analysis, we utilized the “glmnet” R package and trained the model with 10-fold cross-validation to identify the optimal regularization parameter (λ). The best λ value was selected based on the minimum mean cross-validated error [17]. We constructed a SVM-RFE framework with a linear kernel using the e1071 package (version 1.7–16) in R software and the msvmRFE algorithm [18]. A 10-fold cross-validation was implemented, accompanied by an acceleration strategy with halve.above = 100. In each iteration, features with the smallest weights were sequentially eliminated. The model achieved the highest accuracy (0.9167) and the lowest error rate (0.0833) when the number of gene features was reduced to 25. A binary classification gradient boosting tree model was built via the XGBoost package (v1.7.11.1) in R, with objective = “binary: logistic”, max depth = 3, eta = 0.01, and 100 training iterations. Global gain was extracted using xgb. importance, sorted by contribution, and 9 feature genes with importance score > 0 were retained [19]. Subsequently, the VennDiagram package was employed to identify intersection genes from these three algorithms, thus determining the hub genes associated with cell senescence in SPTB.

Expression levels of hub genes were extracted from GSE174415 and GSE118442 datasets from the GEO database. Differences between the SPTB and control groups were compared to identify genes exhibiting consistent expression trends across the two datasets, thereby selecting final hub genes.

### Evaluation of hub genes

ROC curves of hub genes were generated using GSE174415 and GSE118442 datasets via the pROC package in R. Area under the curve (AUC) values were calculated to evaluate the diagnostic efficacy of the identified hub genes and an AUC value > 0.7 was considered to have great diagnostic efficacy.

### GSEA analysis

To identify signaling pathways associated with the hub genes, GSEA was conducted, including GO and KEGG analyses. Results were visualized using the R package ClusterProfiler [14].

### Clinical samples and RT-qPCR

Placental tissue samples were collected from ten women with SPTB and ten women with full-term pregnancies at The First Affiliated Hospital of Hebei North University from January 2025 to October 2025. Written informed consent was obtained from all participants upon recruitment. The study was conducted in accordance with the Declaration of Helsinki and was approved by the Ethics Committee of The First Affiliated Hospital of Hebei North University (approval no. K2024100). Total RNA from placental tissues was extracted using TRNzol Universal Reagent (Tiangen Biotech, China) according to the manufacturer’s instructions. RNA was subsequently reverse-transcribed into cDNA using the FastKing RT Kit (with gDNase) (Tiangen Biotech, China). Real-time quantitative PCR (RT-qPCR) was performed using SuperReal PreMix Plus (SYBR Green) (Tiangen Biotech, China) on the TianLong TL988 Real-Time PCR System (TianLong, China). Relative fold changes were calculated using the 2-ΔΔCT method, normalized to the endogenous reference control gene. Primers were synthesized by Tsingke Biotech (Beijing) Co., Ltd. (Table 1).

**Table 1:** The sequencing of the primers used in this study.

Gene	Primer sequence (5’-3’)
GAPDH-F GAPDH-R TWIST1-F TWIST1-R	GGTCTCCTCTGACTTCAACA GTGAGGGTCTCTCTCTTCCT GTCCGCAGTCTTACGAGGAG GCTTGAGGGTCTGAATCTTGCT

### Statistical analysis

All statistical analyses were performed using R version 4.3.3 (https://www.r-project.org) and GraphPad Prism 8 software. Comparisons between the two groups were conducted using t-tests, with p < 0.05 considered statistically significant.

## Results

### Differential gene screening and PPI analysis

**Fig 1:**
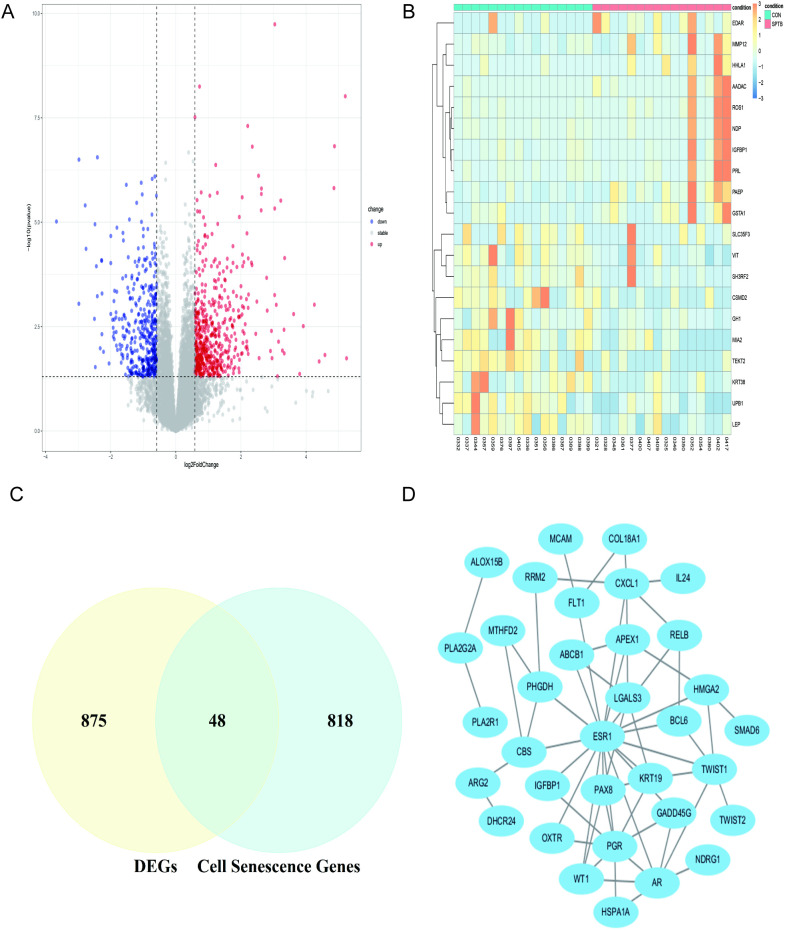
Identification and PPI analysis of differentially expressed cell senescence-related genes. **(A)** volcano plot of differentially expressed genes. Red nodes indicate upregulated genes, blue nodes indicate downregulated genes, and grey nodes indicate genes that are not significantly differentially expressed. **(B)** Heat map of the TOP10 up-and-down differentially expressed genes. The red and blue colors represent upregulated and downregulated differentially expressed genes, respectively. **(C)** Intersection of differentially expressed genes and cell senescence-related genes in a Venn diagram. **(D)** PPI network of intersection genes.

The analysis identified 923 DEGs between the SPTB and control groups. Of these genes, 525 were upregulated and 398 were downregulated in the SPTB group. The volcano plot illustrates the distribution of DEGs (Fig 1A). Heatmap shows the top 10 upregulated and downregulated genes (Fig 1B). Intersection analysis between these 923 genes and 866 cell senescence-related genes identified 48 intersection genes (Fig 1C). PPI network constructed from these intersection genes using the STRING database revealed interactions among 31 proteins, visualized using Cytoscape (Fig 1D).

### Functional enrichment analysis of differential genes

**Fig 2:**
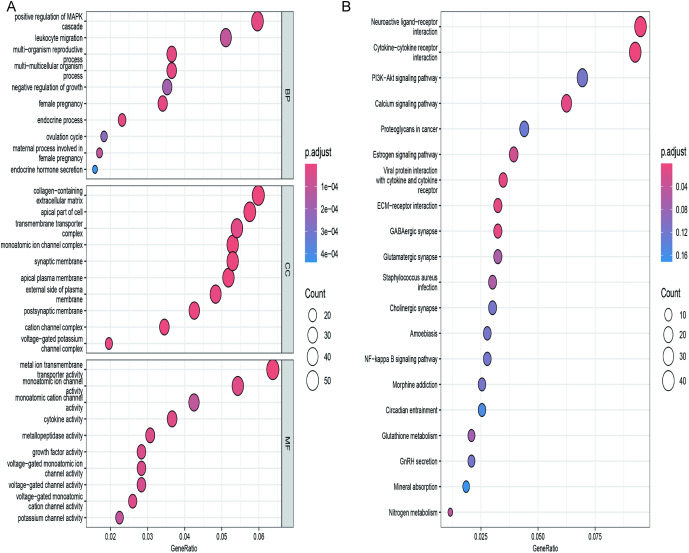
GO and KEGG enrichment analysis of differentially expressed cell senescence-related genes. **(A)** GO enrichment analysis. The bubble plots depict the ten most significantly enriched functions, where the size of the bubbles represents the number of DEGs (the larger the circle, the greater the number of DEGs) **(B)** Analysis of KEGG enrichment, with bubble plots displaying the top 20 most significant pathway enrichments.

To explore potential mechanisms of differentially expressed cell senescence-related genes in SPTB, GO and KEGG analyses were performed. GO enrichment analysis indicated that the 48 intersection genes primarily participated in multi-organism reproductive processes, collagen-containing extracellular matrix, and metal ion transmembrane transporter activity (Fig 2A). KEGG pathway analysis showed enrichment in cytokine-cytokine receptor interaction, neuroactive ligand-receptor interaction, and the PI3K-Akt signaling pathway (Fig 2B).

### Distribution characteristics of immune-infiltrating cells

**Fig 3:**
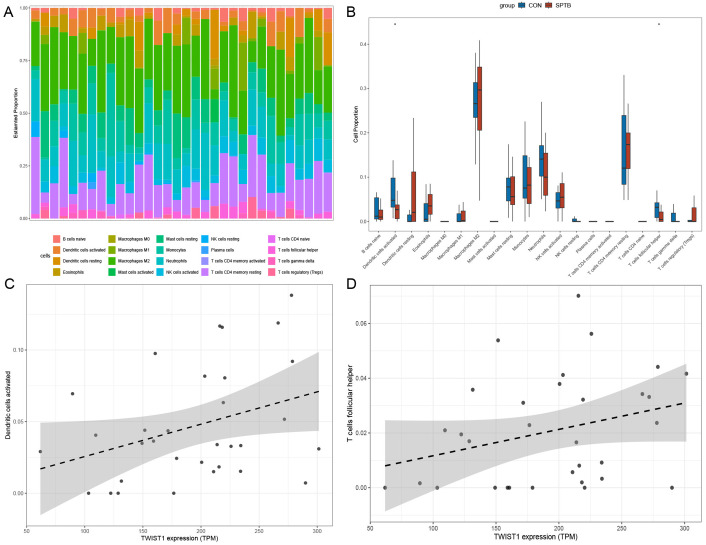
Immune infiltration analysis of SPTB and full-term pregnancy group in the GSE174415 data set. **(A)** The proportions of 20 immune cells between SPTB group and control group. **(B)** Boxplot showing differences in immune cell infiltration between SPTB group and control group (*p-value<0.05). **(C)** Correlation between TWIST1 expression and the infiltration proportion of activated dendritic cells. **(D)** Correlation between TWIST1 expression and the infiltration proportion of follicular helper T cells.

Samples with p < 0.05 were selected using the CIBERSORT algorithm. The infiltration proportions of 20 immune cell types are displayed in the histogram (Fig 3A). Boxplot analysis revealed that the proportions of activated dendritic cells and follicular helper T cells were significantly lower in the SPTB group compared to the control group (Fig 3B). Correlation analysis revealed that TWIST1 expression exhibited a moderate positive correlation with the infiltration proportion of activated dendritic cells (Pearson’s r = 0.35, p = 0.047); however, after Benjamini-Hochberg correction for multiple testing, false discovery rate (FDR) q-value = 0.35. Meanwhile, TWIST1 expression showed a trend toward a moderate positive correlation with the infiltration ratio of follicular helper T cells (Pearson’s r = 0.30), but this trend was not statistically significant (p = 0.097), and after Benjamini-Hochberg correction for multiple testing, FDR q-value = 0.48. Collectively, these findings suggest that TWIST1 may have a moderate positive correlation with the infiltration ratios of both activated dendritic cells and follicular helper T cells, but further validation with an expanded sample size is warranted to confirm these potential associations.

### Identification and validation of hub genes

**Fig 4:**
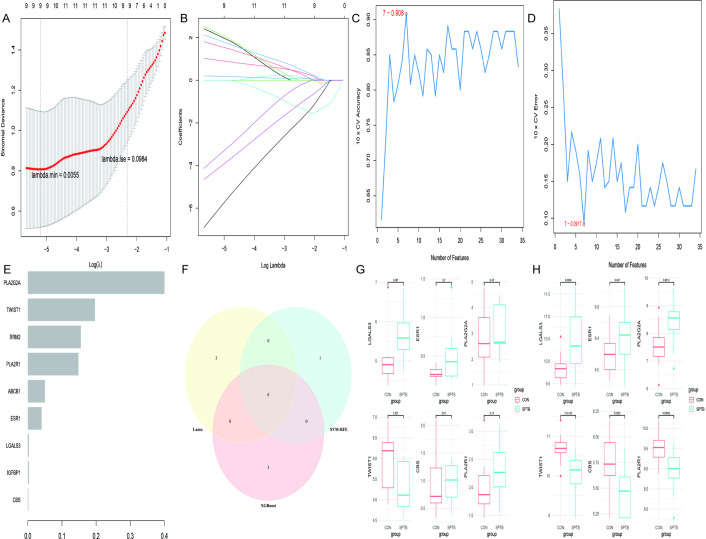
Screening hub genes by machine learning. **(A-B)** The10-fold cross-validation and LASSO coefficient profiles for optimum tuning parameter. **(C-D)** The curve of change in the predicted error and true value of each gene in SVM-RFE algorithm. **(E)** The results of XGBoost algorithms for screening hub genes. **(F)** The venn diagram of the intersection genes screened by the LASSO, SVM-RFE, and XGBoost algorithms. **(G-H)** The expression levels of hub genes in the GSE174415 and GSE118442 datasets.

The LASSO algorithm screened nine potential biomarkers among differentially expressed cell senescence-related genes (Figs 4A and 4B). The SVM-RFE algorithm identified seven genes (Figs 4C and 4D), and the XGBoost algorithm identified nine genes (Fig 4E). Intersection analysis of the three algorithms resulted in six hub genes: LGALS3, ESR1, PLA2G2A, TWIST1, CBS, and PLA2R1 (Fig 4F). Expression levels of these hub genes were compared between the SPTB and control groups using the GSE174415 and GSE118442 datasets. Genes with consistent expression trends in both datasets were selected, ultimately identifying TWIST1 as the final hub gene (Figs 4G and 4H).

### Evaluation of hub gene diagnostic efficacy

**Fig 5:**
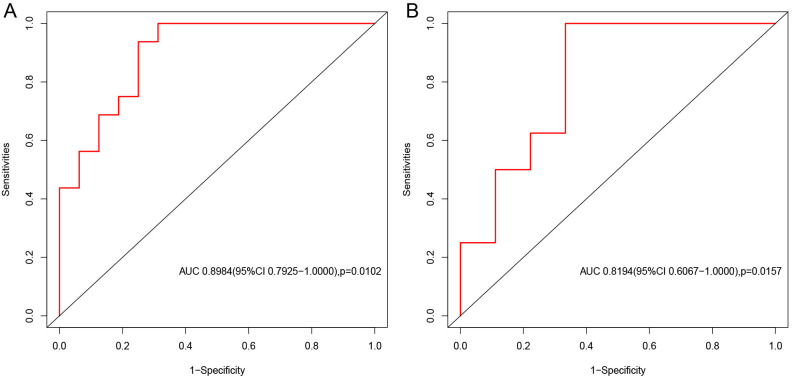
Diagnostic performance assessment of TWIST1. **(A)** The ROC curve of TWIST1 in the GSE174415 training set. **(B)** The ROC curve of TWIST1 in the GSE118442 validation set.

To evaluate the diagnostic ability of TWIST1 for predicting SPTB, ROC curves were generated using the pROC package in the GSE174415 and GSE118442 datasets. The AUC values for TWIST1 in the two datasets were 0.8984 and 0.8194, respectively (Fig 5). These results indicate that TWIST1 is a reliable biomarker with high diagnostic value for SPTB.

### GSEA enrichment analysis

**Fig 6:**
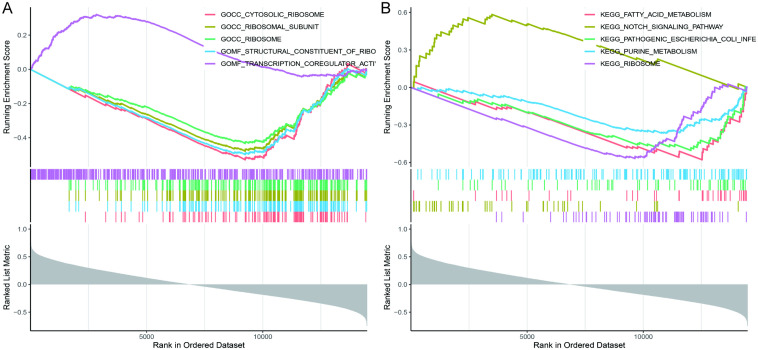
GSEA analysis of TWIST1. **(A)** GSEA plots for GO terms. **(B)** GSEA plots for KEGG pathways.

GSEA enrichment analysis demonstrated that TWIST1 was primarily enriched in GO terms such as cytosolic ribosome, structural constituent of ribosome, and ribosomal subunit (Fig 6A). KEGG analysis indicated enrichment in the Notch signaling pathway and fatty acid metabolism (Fig 6B).

### RT-qPCR validation

**Fig 7:**
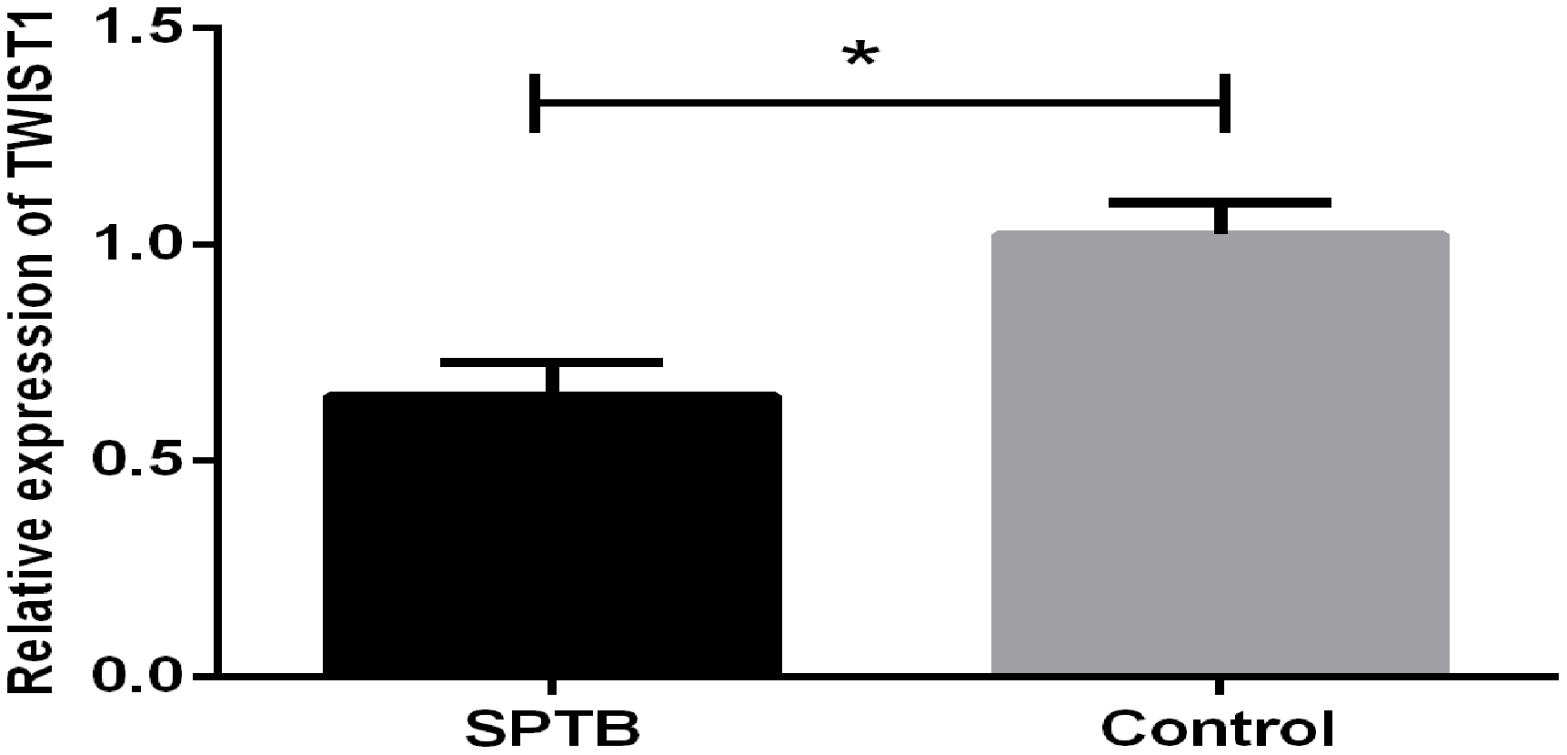
The expression of TWIST1 by RT-qPCR. Relative expression of TWIST1 in the SPTB group and the full-term pregnancy group (control group). (*p-value<0.05).

RT-qPCR analysis showed that the expression of TWIST1 was downregulated in placental tissues from the SPTB group compared with the full-term pregnancy group (Fig 7). This finding was consistent with bioinformatics analysis results.

## Discussion

The etiology of SPTB is not yet fully understood and is considered a syndrome caused by multiple mechanisms. Globally, SPTB is a leading cause of death in children under five years old, and complications associated with SPTB still threaten child survival [20]. It has been reported that cellular senescence can induce preterm birth in mice [21]. Interestingly, accelerated senescence of decidual cells has also been observed in women with preterm birth [22]. Fetal cell senescence induced by oxidative stress injury may trigger delivery [23]. Hypoxia-inducible factor 1 (HIF-1) may induce mitochondrial dysfunction and placental senescence, promoting preterm birth [24]. Cellular senescence and increased inflammation are physiological factors associated with SPTB [25]. We speculate that cellular senescence plays an important role in SPTB, but the specific mechanism remains unclear. Thus, exploring its pathogenesis and identifying related biomarkers remain critical. Through bioinformatics and machine learning analyses, this study aimed to identify key genes related to cell senescence in SPTB and provide insights for future research.

In this study, 923 DEGs from SPTB and full-term pregnancy placenta samples in the GSE174415 dataset were intersected with 866 cell senescence-related genes from the HAGR database, yielding 48 DEGs. Enrichment analysis indicated that these DEGs primarily participate in cytokine-cytokine receptor interactions and the PI3K-Akt signaling pathway. Transcriptomic studies have identified cytokine-cytokine receptor interactions as a common pathogenic mechanism in SPTB [26]. Amniotic transcriptome analysis in the preterm delivery group indicated that up-regulated genes were primarily enriched in the cytokine-cytokine receptor interaction pathway [27]. Previous studies reported that the PI3K-AKT signaling pathway activates the mTOR pathway, inducing cellular senescence [28,29]. This study also examined immune cell distribution characteristics in SPTB patients through immune infiltration analysis. Activated dendritic cells and follicular helper T cells were significantly reduced in SPTB. Immune cells interact at the maternal-fetal interface, maintaining immune balance and ensuring proper pregnancy progression. Disorders among immune cell subsets are frequently linked to childbirth and SPTB, warranting further investigation [30].

Three algorithms were integrated to screen the 48 genes, and a total of six hub genes were obtained, namely LGALS3, ESR1, PLA2G2A, TWIST1, CBS, and PLA2R1. The LGALS3 gene encodes galectin‑3, which can bind to lipopolysaccharide and exert pro-inflammatory effects by promoting the infiltration of immune cells into infected sites [31]. Maternal serum galectin‑3 levels are increased in pregnancies complicated by PPROM [32]. In SPTB, galectin‑3 expression in blood and placental trophoblast cells is elevated, and telomeres are shortened [25]. Telomere length is not only an indicator of cellular senescence; it is also a biomarker of oxidative stress. Oxidative stress can induce fetal membrane aging in PPROM [33]. In women with spontaneous preterm birth complicated by chorioamnionitis, galectin‑3 expression in decidual, chorionic, and placental membrane tissues is increased. Similarly, increased galectin‑3 expression has been observed in decidua, villi, and fetal membrane tissues in SPTB with chorioamnionitis [34]. Elevated galectin‑3 levels have also been reported in preterm births associated with infection [35]. Inflammation can induce cellular senescence, which subsequently triggers the release of inflammatory factors that increase the likelihood of SPTB. Studies have found that ESR1 is essential for fetal survival in preterm mice [36]. Elevated homocysteine levels in pregnant women may serve as a biomarker for predicting preterm birth. CBS, an enzyme in the trans‑sulfuration pathway, is crucial for the endogenous synthesis of cysteine. CBS can induce endothelial cell senescence and increase cellular susceptibility to exogenous homocysteine [37,38]. Therefore, we speculate that altered CBS expression may affect homocysteine metabolism and contribute to SPTB.

To further identify the hub genes related to cellular senescence in SPTB, we selected six candidate genes from the GSE174415 and GSE118442 datasets in the GEO database with consistent expression trends, and TWIST1 was finally identified as the hub gene. TWIST1 was down‑regulated in the SPTB group compared with the full‑term pregnancy group. TWIST1 is an evolutionarily conserved transcription factor belonging to the basic helix‑loop‑helix protein family. It is expressed in embryonic tissues and plays a key role in embryonic development and cell differentiation. TWIST1 has been shown to act as an inducer of epithelial‑mesenchymal transition (EMT), regulating EMT during early embryonic morphogenesis [39,40]. TWIST1 promotes EMT by inducing CDH1/ ECadherin-mediated loss of cell-to-cell adhesion [41]. PPROM accounts for approximately 40% of SPTB. Maternal risk factors can induce oxidative stress and senescence. EMT induced by oxidative stress is often associated with local inflammation, which may occur in amniotic or chorionic membrane cells. Tissue damage caused by such inflammation is considered a harmful factor in SPTB [42,43]. Some studies have shown that TWIST1 is involved in cervical cancer progression by promoting cellular senescence [44]. Senescent cells may play an important role in inducing EMT. The senescence‑associated secretory phenotype of senescent fibroblasts can induce EMT in neighboring epithelial cells [45]. TWIST1 can inhibit oncogene‑induced cellular senescence by eliminating key regulators of the p53‑ and RB‑dependent pathways, thereby inducing EMT and promoting tumorigenesis [46]. Therefore, we speculate that TWIST1 down‑regulation may promote cellular senescence in SPTB, although the specific molecular mechanism remains unclear. According to GSEA enrichment analysis, TWIST1 is mainly enriched in the Notch signaling pathway. Cellular senescence is regulated by multiple complex signaling pathways, including the p53/p21 pathway, the p16/Rb pathway, and the Notch signaling pathway [47]. Previous studies have shown that TWIST1 mediates the Notch pathway to promote esophageal squamous cell carcinoma [48](8). However, research on its mechanism in SPTB is limited and requires further experimental validation. In our study, TWIST1 showed high diagnostic value (AUC > 0.9), providing a theoretical basis for its potential use as a diagnostic marker for SPTB.

We acknowledge several limitations of our research. First, the small sample size may limit the statistical power of our findings. Second, although analysis of GEO datasets indicated an association between TWIST1 and cellular senescence in SPTB, causality cannot be established without functional experiments. Additionally, the use of public GEO data may introduce unmeasured confounding factors inherent in the original study designs. Despite these limitations, our work provides a foundational hypothesis. TWIST1 was identified as a hub gene, potentially serving as a diagnostic marker for SPTB and offering novel insights into the complex biological processes involved.

The primary innovation of this work is the integration of bioinformatic analyses from public datasets with clinical validation, successfully identifying TWIST1 as a promising biomarker and therapeutic target for SPTB. Future research should prioritize functional studies (in vitro and in vivo) to definitively confirm whether TWIST1 modulates SPTB through specific senescence-related pathways, such as the p53/p21 and Notch signaling pathways.

## Supporting information

S1 File (DOCX)

S2 File (XLSX)
