# Supplementary material for: Identification of cell senescence-related genes in spontaneous preterm birth based on bioinformatics analysis and machine learning
Source: PLoS One. 2026 Jan 16;21(1):e0340809. doi: 10.1371/journal.pone.0340809 (PMC12810911; doi:10.1371/journal.pone.0340809)
Supplement: S1 File — (DOCX) [file pone.0340809.s001.docx]

# Data Availability Statement

The datasets generated and/or analyzed during the current study are available in the Gene Expression Omnibus (GEO) repository, under the accession number GSE174415 and GSE118442. The repository contains all relevant raw data needed to replicate the results of the study, including values for statistical analysis.
